# A Psychographic Experiment

**Published:** 1887-08

**Authors:** 


### A PSYCHOGRAPHIC EXPERIMENT.

Many of the readers of HALL'S JOURNAL are familiar with some of the remarkable evidences of spirit presence and control, by means of human instrumentalities, furnished by William Eglington, a world renowned sensitive, who paid a recent visit to St. Petersburg, at the instance of some titled dignitaries of that hyperborean locality. As one of the results of Mr. Eglington's visit, we extract the following account of a sitting, published in a late issue of *London Light*, over his signature :

"The only persons present with Mr. Eglington being the Emperor and Empress, and the Grand Duke and Duchess Sergius. Various experiments were tried, one which has frequently been accomplished, viz.: that of four numbers being demanded in four different colors, the sitters choosing their own colors, succeeding perfectly. Then came the crowning point of this seance. Various answers having been obtained to the questions propounded, the Emperor placed two clean slates together, and he, the Empress and myself held them above the table. Soon the sound of writing was heard, and, on uncovering the slates, one was found to be filled in the hand writing of one perfectly well known to me. I cannot here state what the purport of that communication was, but as it is well known in Russia, as well as to some few in this country, I may at least say that it had reference to an event which occurred a few days after, and which has now become a matter of history. Probably this slate—which is preserved—may in future generations be referred to as a striking instance of the power of the spirit of prophesy. Their Imperial Majesties were much moved by this communication, and a painful silence followed. Luckily, the Grand Duke Vladimir having given into the custody of the Czar a sealed envelope, containing a bank note, I was enabled to break the silence by proposing to get the number written. The Czar placed it between my Brahma-lock slate, the Czarina choosing a piece of red crayon. The slate rested under the hands of the Emperor and the Grand Duchess. After we had heard the writing the slate was opened, and the number 716,990 was found therein. Upon opening the outside envelope the number was found to agree with that of the bank-note. Rising from his chair, and shaking me warmly by the hand, the Emperor said, ' This is truly wonderfnl, and I thank you very much for having been the means of showing me such manifestations.' All were delighted, I most of all perhaps, but saddened somewhat by the events of the evening. and terribly exhausted. Half an hour spent in conversation with their Imperial Majesties terminated this eventful evening, and I hurried off in the small hours of the morning to M. Aksakof's with all the slates with which we had tried our experiments in my possession. These were afterwards distributed to my friends as souvenirs of the occasion.

"With this account of my first seance with the Emperor and Empress of Russia, I must hasten to make reference to other events and seances, lest I tire the reader. As no restriction was placed upon any reference to the sitting above recorded, beyond what was naturally left to my good taste and judgment, I have no hesitation in giving them publicity, but in regard to other interviews I am unable to speak. I may however, say that before I left Russia I was the recipient of a handsome pair of diamond and sapphire solitaires, which I wear in token and remembrance of the events narrated, and because of the honor attached thereto."

